# Trauma Affecting Asian-Pacific Islanders in the San Francisco Bay Area

**DOI:** 10.3390/ijerph14091053

**Published:** 2017-09-12

**Authors:** Pollie Bith-Melander, Nagia Chowdhury, Charulata Jindal, Jimmy T. Efird

**Affiliations:** 1 Chinatown Community Development Center, San Francisco, CA 94111, USA; polliebith@gmail.com; 2 Asian Community Mental Health Services, Oakland, CA 94607 USA; nagiachowdhury@gmail.com; 3 Centre for Clinical Epidemiology and Biostatistics (CCEB), School of Medicine and Public Health, The University of Newcastle, Callaghan 2308, Australia; jimmy.efird@stanfordalumni.org; 4 Center for Health Disparities (CHD), Brody School of Medicine, East Carolina University, Greenville, NC 27834, USA

**Keywords:** Asian Americans, exploitation, immigrants, mental health, refugees, trauma

## Abstract

Trauma is a transgenerational process that overwhelms the community and the ability of family members to cope with life stressors. An anthropologist trained in ethnographic methods observed three focus groups from a non-profit agency providing trauma and mental health services to Asian Americans living in the San Francisco Bay Area of United States. Supplemental information also was collected from staff interviews and notes. Many of the clients were immigrants, refugees, or adult children of these groups. This report consisted of authentic observations and rich qualitative information to characterize the impact of trauma on refugees and immigrants. Observations suggest that collective trauma, direct or indirect, can impede the success and survivability of a population, even after many generations.

## 1. Introduction

### 1.1. Background and Significance

Asian and Pacific Islanders (APIs), as defined by the Asian–Pacific Institute on Gender-Based Violence include “all people of Asian, Asian American, Native Hawaiian or Pacific Islander ancestry who trace their origins to the countries, states, jurisdictions and/or the diasporic communities of these geographic regions” [1,2]. APIs exist in a precarious position as an immigrant group in the United States (U.S.) [3]. Some (e.g., Filipinos) are considered minorities under the law and receive certain social benefits while others (e.g., East Asian and mainland Southeast Asian groups) do not have minority political status and cannot receive benefits despite their poor socioeconomic standings. One group, for example, refugees from mainland Southeast Asia, arrived after 1975 when the U.S. pulled out its military forces from Southeast Asia. Similar to other oppressed communities throughout the world, APIs have been psychologically, emotionally, and physiologically affected by both direct and indirect trauma [4]. Stigma, shame, and the lack of understanding of mental illness, especially symptoms related to trauma, are a major barrier to accessing mental health services. Overall, this population group is least likely to access health/mental health services [3].

Asian Americans, consisting of ~6% of the population, represents the fastest growing API group in the U.S. Out of this 6%, 67% are foreign-born and three-fourth speak a language other than English at home [3]. One common characterization of Asian Americans is that they fall within the pretense of the “Model Minority” myth and appear to live a successful life in the U.S. compared with other minority groups [5,6,7]. Often APIs are perceived to have fewer mental health problems and clinical symptoms than Caucasians [4]. Asian Americans represent the extremes of both health outcomes and socioeconomic status. What distinguishes Asian Americans from other ethnic minority groups, however, is their health-seeking behaviors, particularly mental health services. Approximately 8.6% of Asian Americans sought mental health services compared with about 18% of the general population [8]. This is compounded by the diversity in the Asian population with over 43 ethnic groups speaking over 100 languages and dialects [3], which makes the group as a whole difficult to identify in term of its collective needs.

### 1.2. Conceptual Framework

**Figure 1:**
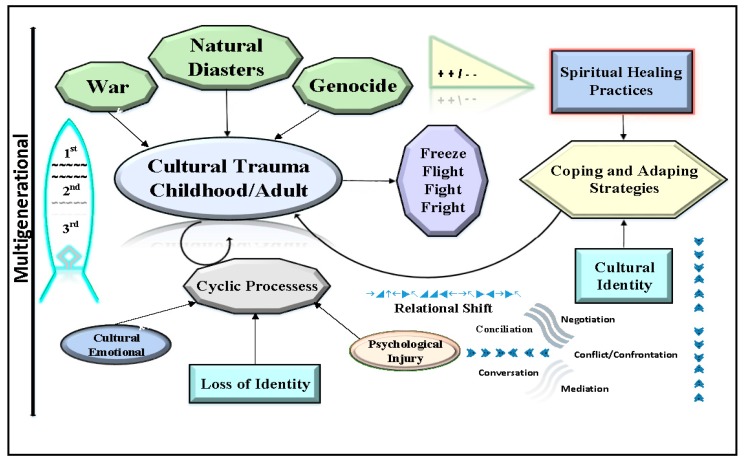
Conceptual Framework for Trauma. Trauma, manifested as freeze, flight, fight, fright, is a complex multigenerational process that juxtaposes conflict/confrontation (war, natural disaster, genocide) with coping and adapting strategies (spiritual healing, cultural identity, mediation, conversation, conciliation, and negotiation). Additionally, cyclic processes such as psychologic injury, loss of identity, cultural and emotional factors play an important role in trauma.

Trauma is defined as having a deeply disturbing or distressing experience, which can include a physical injury. It can affect the social fabric of a nation or culture. The Diagnostic and Statistical Manual of Mental Disorders (DSM)−5’s criterion for trauma is precise. It involves either direct exposure or witnessing in person. Cultural trauma, however, is complex conceptual process (Figure 1). 

It is deeply rooted at the collective level in an event such as a war, natural disasters, or a genocide. Transgenerational or historical trauma is defined as the subjective experiencing and remembering of events in the mind of an individual or the life of a community that passed from adults to children in cyclic processes as “collective emotional and psychological injury” [9]. The link between childhood trauma and adult offending is believed to be the result of unresolved cultural identity and diminished sense of self-worth [10]. Coping and adaptation strategies are simply adopted from one generation to the next. Multigeneration/historical trauma attacks what constitute a culture through the prohibition of the use of language that is in tune with the cultural beliefs and values, spiritual/healing practices, and access to public spaces. It causes a dramatic loss of identity and meaning. Cultural identity and cultural conceptions of reality are evolved, in part, to provide protection against the most basic of all human fears. In other words, people are susceptible to anxiety and related symptoms if they have little faith or lose their cultural worldview [11]. Cultural identity and the cultural conceptions of reality anchors a person’s sense of self and self-worth. Cultural trauma seems to inhibit both of these values and are innately important in the survivability and functionality of a population.

### 1.3. Transgenerational and Complex Trauma (Complex Posttraumatic Stress Disorder)

Transgenerational trauma and complex trauma, also known as complex-posttraumatic Stress Disorder (C-PTSD), have not been well understood until recently [12,13,14]. The general definition of transgenerational trauma is trauma that is passed down from the one generation of trauma survivors to the next, and so on down through the generations of the survivors’ offspring. The latter transmission tends to manifest in C-PTSD. The most obvious evidence in literature on the intergenerational effects of parents’ traumas concerns those individuals who survived the holocaust [15].

Symptoms associated with witnessing or experiencing traumatic events include volatility of emotions, hyperarousal, pervasive fear, and anxiety [16]. It is an adaptive response intended to keep a person away from similar dangerous situations in the future [17]. C-PTSD manifests as a set of symptoms resulting from repeated and prolonged stress of a social or interpersonal nature. Individuals who suffer complex trauma can present with marked emotional dysregulation deficits [17].

The academic literature provides obvious and uncontested evidence of how trauma impacts a community from one generation to the next, and how the additional violence experienced after resettlement in America can adversely affect a community [18,19,20,21]. As noted earlier, traumatic experience is not homogenous even within a specific region. Independent of the diversity among the groups involved, there are some unique patterns that have emerge from specific regions, including from mainland Southeast Asia, East Asia, and South Asia. The early arrival experiences included discrimination and segregation, as well as poor and substandard housing conditions [22]. 

The end of the American/Vietnam War in the mid-1970s brought multiple waves of refugees from Cambodia, Laos, and Vietnam to the United States. Approximately, 1.2 million Cambodians, Vietnamese, Laotians, Mien, and Hmong migrated to the U.S. between 1975s and the 1990s [23]. The first wave of refugees in 1975 consisted mostly of those on the losing sides on the conflict, fleeing from the Southeast Asian communist regimes. Latter waves of refugees from the 1980s and 1990s consisted of those fleeting as a result of political, religious, and ethnic persecution. About 125,000 Vietnamese resettled in the United States in the first wave in 1975. Starting in 1978, another wave of Vietnamese began to flee their country. These were the “boat people,” who were poorer and less educated than the Vietnamese who came in the first wave. These refugee families arrived in the U.S. suffering from PTSD, with limited education, and fragmented English language proficiency. Many of them were vulnerable to poverty, crime, and violence after resettlement in the U.S.

The cycles of violence and trauma continue with recent deportations of refugees from the U.S. to their native countries, back to the very situations and governments that persecuted them in the first place. Many of the refugees being deported were children or infants when they fled Asia, and speak English rather than the language of their native country [24,25,26]. For example, over the past twenty years, more than 13,000 Cambodian, Vietnamese, and Laotian Americans have been served deportation orders, according to the Southeast Asia Resource Action Center [27]. Over 500 individuals have been deported from the U.S. to Cambodia alone since 2002 when the two countries signed a repatriation agreement [28]. As refugees are sent back to countries from which they fled, this U.S. immigration policy is essentially re-traumatizing these individuals.

### 1.4. Cultural Trauma

Cultural trauma refers to an experience that causes a dramatic loss of identity and meaning in the social fabric of a community; it generally affects groups of people who have already achieved some degree of cohesion [11]. The Chinese Cultural Revolution is an example of cultural trauma. Some Chinese Americans are still mourning the loss of identity and cultural values caused by the mass campaign of the Cultural Revolution targeted towards the elimination of Chinese traditional values. Many Southeast Asian refugees also continue to live in an environment of persistent poverty, drugs, and crimes, which creates another form of cultural trauma. Japanese, Filipinos, and other Asians are still living with the past trauma of resettlement to internment camps and discrimination experiences by previous generations.

### 1.5. Evolutionary Perspective

An evolutionary perspective perhaps may offer a unifying and coherent conceptual framework within which etiologies and symptoms of mental illness can be understood. This functional approach facilitates a deeper understanding of how we as human beings react to traumatic events. Trauma impacts us in a fundamental fashion. It transforms the individual on both biological and psychological levels. The general biological processes underlying the stress response are said to be universal; however, the specific dynamics are a function of the unique sociocultural environment and psychological makeup of the individual [29,30]. Fear is the key emotion in PTSD. Fear is an evolved function which serves as a motivating survival trait through defensive behaviors [31,32]. It has been suggested that fear may be a defensive option taken to the extreme, part of the functional adaptation of humans to dangerous environments. Evolved mammalian defensive mechanisms can be characterized into six underlying components: avoidance, attentive immobility, tonic immobility (TI), withdrawal, aggressive defense, and appeasement. These six defenses have been selected early on in evolution, as humans were vulnerable to various predators [33]. For example, several other writers have suggested continuities between TI (as seen in “playing possum”) and the dissociation that sometimes accompanies trauma. However, it is important to differentiate between the two types of immobility which may be essential for defense: attentive immobility, which makes us stop and use all of our senses to identify a threat, and TI, which goes further in the face of an overwhelmingly dangerous threat [33]. Some of us freeze (or become immobile) when confronting dangers. Thus dissociation, which is commonly understood as pathological, may have been a defensive option taken to the extreme, and part of a functional adaptation to dangerous environments [33].

Recent contributions to evolutionary theory on numerous psychopathologies offer insight into human past experiences with dangers. The anxiety spectrum, for example, is rooted in the way the human species responds to danger. Evolutionary study is about looking backward to understand the traits that have survived through many generations by serving an evolutionary purpose. This is not to suggest that traits that are still present continue to provide advantages to the species. However, certain characteristics or traits, such as phobias, might have roots in the way we as humans once survived or recognized danger. As some authors have suggested, “evolution is not forward looking and could not anticipate a future where being stared at by a large group of nonsmiling, non-kin specifics was more likely than not to be followed by negative consequences” [34]. However, evolutionary theory offers sound scientific explanation of why certain human behaviors are present and what happens when too much of such behaviors are displaced. In words, it becomes problematic when there are too much of these behaviors. All biological phenomena, including human basic emotions, are considered to have evolutionary advantages. Two separate kinds of explanations has been offered in order to understand human responses to emotions. The first type involves a proximate explanation of the structure, regulation, and ontogeny of the glow organ [32]. The evolutionary explanation accounts “for the function of the character, its evolutionary history” and why any emotion exists. The benefits of having emotions stems from broad categories of functions. These functions lie in the areas of motivation, communication, and cognition [32]. Emotions are defined as specialized modes of operation that are shaped by natural selection in order to adjust the physiological, psychological, and behavioral parameters of a species in ways that “increase its capacity and tendency to respond adaptively to the threats and opportunities characteristic of specific kind of situations.” It is general knowledge that certain kinds of situations arouse certain types of emotions and that natural selection shaped the various emotions [32]. Some of the basic responses such as fear, panic and agoraphobia and moods (such as sadness and happiness) have evolved to serve specific functions for organisms, and especially in human beings [29]. The anxiety spectrum, from anxiety to PTSD, can best be explained using this perspective because these traits are found not only in our species but also in the animal kingdom. 

### 1.6. Freeze, Flight, Fight, and Fright

The concept of freeze, flight, fight, and fright can be explained using an evolutionary framework. Our response to danger has its roots in human evolution as part of the defense strategy. This lies at the core of involuntary functions in the human brain. We are wired to record potential risks in order to protect ourselves from danger. Various research studies have focused on the concept of TI in species, including humans, as related to evolution in an attempt to gain a deeper understanding of our responses and reactions to danger. An evolutionary framework helps explain the various ways we confront danger. Some of us run when we feel afraid, some of us freeze when we face danger, and some of us fight when we perceive that we are at risk of being hurt or killed. There is extensive scholarly literature on TI that attempts to explain why such a response is necessary and how it evolved in all species over time.

TI is described as a basic defence strategy that every species has built in its involuntary response in the brain. It has been proposed that TI may allow a species an opportunity to perceive and find a way out [35]. In the case of TI, a species is said to respond in a non-reactive and immobile manner because it perceives that escape is not an option. There is no way out of the situation and thus a species assumes its best course of action is inaction. Data seems to suggest that TI may be a relatively frequent phenomenon in victims of rape and sexual abuse, but its occurrence has not been systematically explored in other types of trauma [35]. Research conducted on a selection of individuals who had experienced various types of trauma, from sexual to violent, specifically examined TI. This retrospective study used a sample of 100 university students in order to understand whether TI varies with the type and nature of the trauma experienced [36]. Participants were asked to define the levels of severity and types of trauma. The TI Scale was used to measure immobility and trauma was assessed using the modified Traumatic Events Questionnaire. The study results found that 70% of the sample had experienced some kind of trauma; no significant differences in TI were noted between different types of trauma (e.g., physical abuse, assault or aggression, and serious accident). The study did find that the mean TI score was significantly higher in the group with trauma that was directly physical/psychological or related to sexual abuse than in the group that indirectly experienced trauma from receiving news of the mutilation, serious injury, or violent or sudden death of a loved one. The tentative conclusion is that TI may not only be typical of sexual traumas but also of other kinds of directly experienced trauma.

The fight or flight response is a physiological response that is triggered when a species feels fear. Fear is a normal emotional response in species to a perceived threat or danger. Fear is also closely associated with anxiety in some ways [34]. The fight or flight response is best explained in evolutionary terms; however, it is solely based on a functional approach. This reaction evolved to enable species to react with appropriate actions to either run away or fight. Within the evolutionary framework, the emotion of fear protected us from dangers, predators and other threats, and thus served to help the species survive. Fear serves as a form of protection against predators/dangers; therefore, fear is adaptive, functional, and necessary [37]. There is also another important aspect of fear that has to do with decision-making processes as well as survival. When an emotion is triggered, it impacts how we make decisions in certain situations. One research study examined risk-taking in order to understand how we react and make decisions when confronted with emotions such as fear in human beings. The study found that individuals with certain personalities react to fear in more negative ways, and that those who experience a more extreme emotion of fear tend to perceive risk at a higher or more severe level. They also found that participants who were fearful consistently made judgments and choices that were relatively negative and pessimistic, and those individuals tended to amplify their perception of risk in a given situation. This is in contrast to participants who were happy or angry; both those groups were more likely to disregard risk by making relatively optimistic judgments and choices [37]. In addition, individuals who had personality characteristics dominated by the emotion of fear, tend to avoid taking risks that are generally perceived by others as relatively nonthreatening [38].

### 1.7. Mental Health Problems and Treatment

Asian Americans seem to respond best to mental health professionals who have similar backgrounds to themselves than to Western mental health professionals who do not share their cultural, ethnic or linguistic backgrounds. Asians are not a homogenous group, and because they have diverse historical backgrounds, providing mental health services is often complicated by the fact that there is not a single model that fits all. The Asian population in the U.S. has more than 43 ethnic groups speaking over 100 languages and dialects [3]. Therefore, it is difficult to identify and collectively respond to patterns of mental illness or to develop prevention interventions that can meet collective psychiatric and psychological needs. In addition, Asian Americans have a long tradition of maintaining one’s reputation, which can affect mental health treatment. The concept of saving face can make it difficult to acknowledge the mental illness of family members and the likelihood of seeking mental health services. In order to understand the Asian American community’s response to mental health problems, it is important to examine the interconnectedness of health-seeking behaviors with cultural values as well as migration experiences and post-migration resettlement situations in America.

The fact that such a low number of Asian Americans actually seek mental health services compared with the general U.S. population may in part be attributable to the idea of shame and stigma that runs deep in Asian American cultural values, beliefs, and practices. This value or belief tends to hinder one’s ability to seek mental health services. In other words, Asian Americans would rather avoid seeking help in order not to deal with stigma or shame.

The experience of Asian Americans in America is similar to that other ethnic minorities in that they have been subject to poor treatment, discrimination, and racism. There has been anti-Asian racism since the first Asians arrived in America, and laws passed to deny basic rights, from marriage to property ownership to citizenship. By the 1900s, for example, the Alien Land Acts and Chinese Exclusion Act were passed to exclude land ownership and bar Chinese from entering the U.S. 

Migration experience provides useful information in examining the root causes of psychological/emotional problems in a population. Again, the refugee/immigrant experience has been postulated to consist of a pre-migration, migration, encampment, and post-migration phase [22]. Each of these periods is associated with different stressors. We are better able to understand how Asian Americans cope with their new realities in the U.S. by examining these different stressors. Language barriers, culturally sensitive mental services, and difficulty of accessing public services generally are compounded by other issues (e.g., psychological or psychiatric problems, Western treatments, lack of family support systems, and financial shortfalls that can precipitate mental health crisis among vulnerable individuals.

Recent groups arriving to the U.S. from Southeast Asia and elsewhere on the Asian continent have suffered the psychological and emotional effects of recent wars in their homelands. They often struggle to assimilate into mainstream America. Many have undergone torture, psychological and physical trauma, separation, and loss of family members. More than half of Southeast Asians have serious, often chronic, physical disorders caused or exacerbated by a mental health disturbance. Some of these conditions apparently were caused by pre-immigration trauma, including torture [39]. Some of them have shown or manifested significant levels of PTSD, general anxiety, and depressive symptoms.

Some Asian Americans continue to struggle to accept the Western modality of mental illness, and to understand what services are available to them, how to access these services, and recognize the symptoms associated with severe mental illnesses such as PTSD, schizophrenia, and bipolar disorder. Because of this lack of understanding, stigma/shame has persisted and continues to play a major role in the ability to decide to seek help.

The focus of this paper is on how trauma impacts a community from one generation to the next and what happens to a community that continues to be affected by violence after their resettlement in America [8,40]. In particular, Asian Americans’ experiences are highlighted with an attempt to understand how trauma (both direct and indirect) is impacting their overall health and well-being. The manuscript also addresses which segments of Asian Americans are most vulnerable, and what lived experiences have helped them to cope or adapt to trauma or have hindered their abilities to seek help. Finally, it outlines what mental health services are appropriate for this population.

## 2. Materials and Methods 

### 2.1. Secondary Sources

This paper utilized information collected from 15 staff members working at a non-profit agency providing trauma and mental health services to Asian Americans living in the San Francisco Bay Area of California in the U.S. The clients of the non-profit agency were predominantly Chinese, Vietnamese, and Cambodians.

### 2.2. Field Observations

An anthropologist (PB-M) trained in ethnographic methods observed three focus groups from the same agency [41]. Between 5 and 10 individuals participated in each group and each session lasted approximately one hour. The participants discussed their experiences relating to trauma and how best to heal themselves. Observations from these groups took place between January and March 2016. All participants were told of and agreed to the observer’s presence during these meetings. 

### 2.3. Methodology

A point of reference strategy was used for observing clients who received mental health services from the non-profit agency [42]. This included secondary information from staff interviews and notes from at least three group observations. Ethnographic methods and standard anthropologic techniques were used to summarize observations.

## 3. Results

Participants ranged in age between 5 and 60 years old. In total, 28 clients were in three group observations (18 Cambodians and 10 Chinese) plus staff notes for 1029 clients. Out of the 28 observed clients, 17 were male and 11 were female.

### 3.1. Group Observations Overview

#### 3.1.1. Group One

All the Cambodian women in the first group were very engaged and all volunteered to check-in and discussed all daily challenges. Three women discussed physical symptoms and one woman attributed her psychological condition to diabetes. Four men in the first group said they did not feel medication support was necessary. One man in the first group said he had a mental disability and health problems owing to the war and violence he experienced. Two of the participants said they were happy to be divorced. One said she would never remarry. Three of them had issues with authority. One man said his physical ailments are as a result of the Pol Pot regime. One woman said she was married in a refugee camp and divorced in the U.S. She has four children, one of which attends university. She wants to move to another city, but she is afraid of not knowing how to get around town. One man said he is concerned about his housing situation and high rent. He said he cannot afford such a high cost and wants to get a job. He said he cannot work owing to emotional problems. He attributed his psychological conditions to the Pol Pot regime and the American/Vietnam War.

#### 3.1.2. Group Two

In the second group, seven females volunteered to check-in. They made jokes about their psychological conditions being the result of trauma in the Pol Pot regime. One man said he needed help with translation because he wanted to tell the psychiatrist to switch his medications. He said the medication that he was taking had serious side-effects. He wanted to cry all the time. He was anxious and could not sleep; he hated everyone and had a short temper. Another man said he needed help with a Medi-Cal application form and making a doctor’s appointment. One woman wanted to stop coming to the support group because she wanted to take cosmetology classes. Another woman giggled and then said she was enrolling in a hair-cutting class for beginners. She said she did not want to take her medications anymore because they made her feel crazy. She said she could not think or behave normally; she felt anxious all the time. One woman said she became violent and frustrated if she did not take her medications. She had violent thoughts toward people around her. She said she had nightmares almost every night; she had dreams of the Pol Pot regime. In her dreams, she saw people dying. She saw dead bodies everywhere. She was hungry. She cried. These nightmares were repeated every night, sometimes more vivid and more violent. She said she woke up feeling sad and sick in her stomach. She sweat profusely when she woke in the middle of a night. She felt alone. She said her husband does not understand her and she could not talk to him about it. She said he gets scared if she tells him about her dreams. One man said he forgot where he was going on a bus after he fell asleep. He said the bus driver had to call police and ask for assistance. Another woman echoed the same sentiment and said she too was lost after she woke up one time while she was on the bus. She was completely lost and had lost her orientation. She could not remember where she lived or whether she had a family. She said there was nothing in her head. Two women said they lost their conscience after they took their medications. They forget how to do the most basic things like cooking or bathing. Another woman said she usually gets disorientated after she took her medications. She lost her sense of self and her sensitivity toward the feelings of others. She said she felt sad and she cried a lot. One woman said she was hungry all the time, especially after she took her medications. She said she was gaining a lot of weight and she was worried. One other woman said since she was diagnosed with psychosis, she has not been the same. She hears voices telling her to do bad things. She said she does better or thinks better after she takes her medications. 

#### 3.1.3. Group Three

There was no conversation in this group, but one man was actively engaged in Chinese calligraphy. He did not look up at the observer (PB-M) when she entered the room, but the rest of the participants did look up. They did not show any reaction and soon they returned to work on their respective projects. Soft Chinese music played in the background. No one talked at all. 

### 3.2. General Findings

The general finding from the group observations are organized into the following categories: (1) Migration experience; (2) Western modality, treatments, and side-effects; (3) Symptoms and experiences related to war; (4) Daily challenges and barriers; and (5) Healing through art.

#### 3.2.1. Migration Experience

The majority of clients had similar migration experiences of war trauma during the Pol Pot regime, violence in the refugee camps, and violence in their neighborhoods in the U.S. They all discussed their experiences of feeling fear of witnessing loved ones being hurt or tortured during the war and/or the pre-migration process. They talked about the difficulty of leaving their homes, personal belongings and other family members behind when they left their country. While in refugee camps, some were abused or physically assaulted by guards or trapped in cycles of violence instigated by different political groups. They are now also constantly faced with violence in their neighborhoods after resettlement in the U.S.

These stories have been often discussed in the group. For example, when one participant wanted to share her story about her life before coming to the U.S., another participant, a male in his 30s, said, “We heard your story many times already.” The female participant responded with a giggle by stating, “I know I shared already.” She plunged ahead with her story. She said she was married in a Thai refugee camp and was later divorced. She gave details about her children; she was very pleased her youngest was attending university. She said her husband left her after they came to the U.S. to go to Cambodia and remarry a younger woman. She said her journey to the U.S. was similar to other Cambodian refugees’ journeys. She said she started walking from her home village and stopped at many places in transition refugee camps in Thailand, then went to the Philippines, and settled in her final destination in the Midwest. Later she moved to the San Francisco Bay Area after she made contact with family members in Cambodia. She said one of her cousins told her about Cambodians and family members living in California. She added that California seems less like a foreign land, but the Midwest did feel strange. She also stated that California weather resembled Cambodian weather and this was the deciding factor in her relocation. She would be better off, so she packed up the family and moved to California after one year braving the cold climate in the Midwest. 

Another participant, a female in her 40s, echoed similar experiences of migration. She was sent to a refugee camp in Thailand at first, then to a camp in the Philippines. In the Thai refugee camp, she was asked by officials to choose what country in the West she preferred to resettle. She was given four options: Canada, Australia, Japan, or the United States. She said she did not care where she was relocated, but she learned that people spoke French in Canada. She chose the U.S. without any specific reason. She said she was happy to leave the refugee camp. The two participants spent a good portion of the time discussing their migration experiences. The rest of the members generally agreed and nodded their heads. 

#### 3.2.2. Western Modality, Treatments, and Side-Effects

The majority of the clients struggled to grasp with what it means to live with psychological problems and treatments for those problems that are often not well understood. The clients came from a different belief system for treatment and care in which the mind-body connection is key to understanding symptoms and symptom management. At the same time, they perceive Western medicine as something similar to magic that cures symptoms, which is contradictory to the Western treatment of mental illness, which uses treatment to minimize symptoms (for example, by daily ingestion of medications) but does not offer a cure. 

Most participants spoke about struggling to cope with symptoms and medications. Some participants did not understand their diagnoses and why the medication seemed to cause more problems. The participants complained of insomnia, loss of appetite, nausea, jittery feelings, and fatigue from the medications they are taking. There also had questions about health care services and the limitations of that care. 

One female participant said, “I think too much and it hurts my head.” She said she cried a lot and was feeling sad all day. She could not make herself feel better. She said if she were happy, she would be okay to go out and do things. 

Another participant, a middle-aged female, stated that she was forgetful and at times could not remember where she lived or where she was going. She also said that she did not like to take her medications because they made her feel like she lost her sense of self; they made her feel numb, and as if she was having an out-of-body experience. She might need to change the dosage to improve her *arom* (“consciousness”) or *arom chhit* (“heart’s consciousness”). This particular participant felt that the medication she was taking made her lose her sense of self and who she was (i.e., consciousness). She said she was unable to think clearly and felt as if a big cloud were hanging over her head.

Similar, another participant, a young female, broke the silence and began talking. She said she did not like her medications because they made her sick. She wanted to stop taking them. She said she could not feel the left side of her body. She had insomnia. She said she felt disconnected from herself. Her mouth became dry from the medications. She said she felt panicked, anxious, and afraid of everything. She said whenever she took the prescriptions her heart rate increased and she lost her ability to think.

Another participant, a female in her 40s, stated that she could not eat or sleep. She felt cold all the time. She wanted to know if her diabetes was giving her problems. She had been diagnosed with type II diabetes and wondered why she was always cold. She said she did not like taking the medications. She wanted to stop taking them. She was worried that she would gain too much weight and get fat. She said she had problems with weight gain after she started taking the medications that her psychiatrist prescribed to her.

#### 3.2.3. Symptoms and Experiences Related to War

The majority of the members in both Cambodian groups described their symptoms as something that was caused by the Pol Pot regime. They stated that they believed that had they not experienced atrocities, they would not be psychologically impaired. They expressed that the violence, the guns, and killings did something to their brains. Some said they had felt numb since then when they witnessed someone being killed or hurt during the war. Others said they felt overly sensitive as a result of witnessing a loved one being hurt or killed.

For example, one participant said she experienced increased anxiety when she thought about or tried to leave her home. Another one said she did not like leaving her house for fear of gun fire and other related violence in her neighborhood.

Another female participant said her problem began after resettlement in the U.S. She wanted to socialize with other people, but she did not want to leave the house when she felt sad and was crying. Her eyes would show she had been crying and she said she would be embarrassed to interact with other people in that state. She also stated that when she felt sad, she got angry. She attributed her psychological conditions to the war in Cambodia. When she got angry, she got violent. She would throw things, such as sticks, shoes or bags, at people. She was still trying to overcome her anxiety, fear, and panic attacks that occur whenever she witnesses a violent event or hears the voices of people fighting from the window of her home. She said she lives in a violent neighborhood. Her neighbors scream and yell at all times of day or night.

Another female participant stated that the war in Cambodia had done things to her surviving family. She attributed her son’s psychiatric condition to her own psychological problems. Her only child tried to commit suicide by cutting himself. He was diagnosed with schizophrenia when he was very young. She said he never recovered from the war and experienced ringing in his head and heard voices. Everything frightened him, including the outside world. She said she felt hopeless and helpless, but she was hoping that the doctor could save him. She had him committed to a hospital and then he was referred to a more permanent facility where doctors could monitor him more closely. The group offered sympathy and acknowledged how brave she was to have to go through seeing one’s son go through such an ordeal. They took turns stating, “Thank you for sharing your stories.”

Another middle-aged female participant said she had been unhappy lately, but she had no reason. She too was affected by violent past experiences. She stated that she wanted to see her children, but they lived too far away in another city; she said she felt lonely now that she lived by herself. She said that her family believed she was never right after she left Cambodia. She witnessed some disturbing events that changed her life. Her violent experiences did not take place during the Pol Pot regime, but afterward in the refugee camps where she was abused and tortured by Thai soldiers. She had also been trapped in battles between Thai soldiers, guerrilla fighters, and Vietnamese soldiers numerous times when she and her family tried to leave Cambodia. After she stated this, the group’s mood then changed. No one talked after that. The facilitator allowed them time to contemplate in silence. It was at this moment that one older male participant began laughing. He spent the next thirty minutes talking to himself. He appeared completely disengaged from the group.

The same man who had disengaged from the group began talking intermittently. He too appeared to be affected by the war. He laughed first and then he cried. He said something inaudible. Then he cried out loud, “Shoot them! They are coming!” He had not engaged with the group, but it was obvious he had symptoms of PTSD. He also appeared manic. Soon he had another outburst. He was singing this time. The facilitator looked up at him and tried to engage him, but he did not seem to notice her. She called his name several more times, but he ignored her completely. Soon the disengaged man had his third outburst. This time he was clearly audible. He said, “Bananas, gardening, and planting. Shoot them! Put up your gun!” He then giggled to himself. He did not look at any participant in the group. The facilitator tried to ask what he was laughing about, but he again ignored her completely. Then he had another outburst. This time he said, “Take out your guns! Why are not you shooting? He is the enemy!” It appeared that he was reliving past experiences or hallucinating about atrocities during the war in Cambodia. After that, the facilitator tried to engage him again, but she failed. He became incoherent and began talking to himself again. 

At the end of the session, I asked the facilitator the reason for the participation of the disengaged man, because his symptoms seemed more severe than that of the others. She said that the group members wanted him with them. They had all migrated to the U.S. at about the same time and lived in the same neighborhood. They said he had been better before for a while, but had regressed recently. I also noticed that one of the participants, a male in his 50s, gently took the man’s hand and led him out of the room at the end of the session. There was a clear bond of understanding among the group. When they all walked out together while chatting loudly and lively, the quiet disengaged man was included among them.

In another example, a client attributed her difficulties to past war trauma. Another female participant said she gets sad easily and wants to cry. She said she thought too much about everything, the past and present. She talked about her experience in the Pol Pot regime and how she believed hard labor had impacted her ability to think clearly now that she was older.

The oldest male in the group concurred with her, in yet another example. He said, “It’s obvious that the Pol Pot regime did things to the people’s heads, even now.” He said he was generally frightened of loud noises and the sounds of airplanes. He stated that he was diagnosed with PTSD after he lost his job and became depressed. He said if he knew how to speak English, his life would be better. He attributed his inability to learn to being in the Pol Pot regime and the experiences of war and fighting thereafter, and said he felt constant fear of violence in his own neighborhood. 

#### 3.2.4. Daily Challenges and Barriers

The group members were concerned about some of the challenges they face daily. This included using public transportation, making doctor’s appointments, and filling out forms for government services. Several members in the group talked about their children and how lonely they felt now that they were grown up and living their own lives. Some of the discussion focused on loneliness and isolation and what to do with their free time; housing situations or the lack of affordable housing; and being divorced. When discussions of daily challenges came up, participants often began talking at the same time, along with side conversations.

For example, one participant stated she wanted to move. She said she had a Section 8 voucher. An older male participant stated that he would like to move out and find a three-bedroom apartment, so that all his children could move back home. He emphasized how he wanted to have a big family again. Another younger participant said that public assistance income was not enough to support him. He needed to work, but he could not seem to get himself to apply for a job for fear that he would have panic attacks at work and get fired.

One participant, one middle-aged female, cried in the group. She said it was difficult to be in this country. She said she does not speak English and had a hard time getting around. She felt lonely and isolated. She wanted to move to another city to live close to her children, but she could not go. She said she would have a hard time looking for a new doctor there and getting around town.

#### 3.2.5. Healing through Art

One recovery and rehabilitation group focused on healing through art rather through speaking and group interaction. The healing through art group was meant to help participants gain independence and a sense of self-worth as they learned to cope and manage their own psychotic symptoms. The clients observed in this group were considered as having severe mental illness and were all diagnosed in the schizophrenia spectrum. None of them spoke English. This support group was meant to provide them with an outlet to heal and express themselves through art.

More males than females attended this session. The facilitator spoke both Mandarin and Cantonese, but only interacted with members when asked to do so. Otherwise, everyone worked quietly on their own projects and did not interact with one another. This particular group focused on arts as a form of rehabilitation and recovery. All members worked on individual projects. All had been diagnosed within the schizophrenia spectrum. This was the only criterion for participating in this art group. The group was aware of the observer’s presence and consented to her observations. However, the facilitator did not expect that any of the group members would interact with the observer.

A few of the projects stood out immediately. One middle-aged male member worked on a collage made of pictures of singers, movie stars, and famous politicians. The collage included some pictures of what appeared to be of his own family members. He worked diligently and rarely looked at others in the group.

Four of the members painted pictures of flowers and cherry blossoms. They chose different colors. Another man of retirement age worked on Chinese calligraphy. He worked in a delicate manner with slow strokes and deliberate moves. He wrote on a huge piece of painting paper. The facilitator said quietly that this male participant also wrote poems. He remembered hundreds of poems, including old poems that had profound meanings. The facilitator mentioned that he was trying hard to manage his psychotic symptoms. For example, he recited these poems constantly to control the voices in his head. He did calligraphy at the group, at home, on the bus, in the streets, and at the playground. Wherever he went, he took a piece of paper with him. He rarely said much, according to the facilitator.

The members worked quietly the entire session. This group lasted about three hours. No one seemed to notice the others in the room. Except for soft Chinese instrumental music heard in the background, the room was generally quiet. They all worked in isolation and only looked up occasionally.

### 3.3. Secondary Findings

#### 3.3.1. Client Demographic and Diagnoses

Information presented in this section were drawn from staff notes and interpreted in terms of client demographics and diagnoses. These notes came from the clinic, one of the agency’s three departments (Clinic, Developmental Disabilities, and Outreach/Advocacy). Therefore, the numbers below refer only to the clinic and are not indicative of the total number of clients served by the agency as a whole.

#### 3.3.2. General Findings

After reviewing various notes on the agency’s clients, one major theme stood out with regard to Southeast Asians. The majority of the clients are suffering from anxiety spectrum (e.g., generalized anxiety disorder, social anxiety disorder, PTSD, panic disorder, acute stress disorder, and some other mixed features) as a result of their pre-migration, migration and post-migration trauma. Most of them live in violent neighborhoods in an urban city. Some clients experience great difficulty after enduring violence and harsh treatment in their native countries and/or while fleeing as refugees. After resettling in the U.S., they continue to feel fear in this foreign land.

The general findings regarding aspects of trauma can be organized into the following categories: (1) Rates of PTSD and diagnosis complications; (2) Trauma manifestation and perceived psychological control; and (3) Trauma, stigma and daily challenges.

##### Rates of PTSD and Diagnosis Complications

The clients in this agency have a considerably higher rate of PTSD than the general population rate of approximately 3.5% [43], perhaps owing to their migration experiences of escaping some of the most brutal regimes in history [44]. Clients from Burma and Cambodia, for example, appear to have a higher rate of C-PTSD and trauma-related conditions compared with those individuals who did not live through such brutal regimes. However, the higher rate of PTSD was similar to other published report pertaining to the immigrant/refugee population [44].

Most of the clients in the agency experience trauma repeatedly for months or years at a time during different phases of migration. The current PTSD diagnosis does not always capture the severe psychological harm that occurs with prolonged and repeated trauma. Some clients at the agency have lived through concentration camps or have been prisoners of war. Some were tortured repeatedly while in captivity. Those who have experienced prolonged totalitarian control with organized violence either in a political, criminal or domestic context, exhibit psychological problems more severe, more complex, and more enduring than the current definition of PTSD found in the DSM-5 criteria.

Overall, clients did not feel that it was relevant if they knew of their diagnoses or the name of their psychological conditions as described by the DSM-5 criteria. Of fundamental concern is how these disorders are being translated into other languages and cultural contexts for these clients. A diagnosis such as bipolar disorder, borderline personality disorder, obsessive-compulsive disorder, or schizoaffective disorder—is often lost in translation, both in the meaning of the words and in concept of the Western model of mental illness. The issue lies in the etic versus emic conceptions of illness. The etic conception stems from a Western modality whereas emic interpretation is innately indigenous. These two views of illness are often incongruent or contradict each other. 

The emergent themes in DSM-5’s diagnoses that are most relevant here are those associated with trauma and trauma-related experiences. Such attention given to transgenerational and complex trauma with its own spectrum in the DSM-5 could provide some relevant significance to understanding how trauma affects an individual and an individual’s responses and reactions to repeated violent events. Diagnoses that are rooted in these violent events such as PTSD, generalized anxiety disorder, major depressive disorder with mixed features (i.e., anxiety or mood disorder), or adjustment disorder with mixed features (i.e., anxiety disorder or depressed mood) may offer us insight into our ability to survive in time of confronting with threats and our ability to cope with the consequences thereafter. 

##### Trauma Manifestation and Perceived Psychological Control

Trauma impacts an individual for a very long time and can pass on from parents to children. It was not uncommon for Chinese clients who were diagnosed with schizophrenia to hallucinate with symptoms that appeared rooted in the Chinese Cultural Revolution. Some of the clients were born in the Bay Area and did not have direct knowledge about that period, but nonetheless described their lived experiences in vivid detail, vis á vis the transgenerational passage of fear and trauma from close family relatives and friends. The Cultural Revolution was a complex social upheaval that began as a struggle between top party leaders. Generations of Chinese who lived through that period felt that their culture was stripped away and replaced by a foreign concept and political system. 

Similarly, some of the Cambodian clients still feel that they are being watched by communist leaders. One older man seemed to lose his ability to be present and engage with the other group members. When he blurted words out loud, some of those utterances were innately war-based. One particular example occurred when he yelled out loud, “Shooting each other! Call off fire!”

Many Burmese, Cambodians, Hmong, Mien, and Vietnamese clients were tortured or lived in an environment in the refugee camps with the constant fear of being physically harmed by guards, soldiers, and other authorities. This form of torture is a deliberate infliction of severe physical and mental pain and suffering. While some experienced torture under their own governments, others suffered abuse under the governments that ousted the communist regimes. Also, some clients suffered atrocities committed by people who were their neighbors and friends living in their communities back home. This type of experience has created for clients a distrust of strangers and difficulty in relating to others. Some clients have become paranoid and sensitive to sharing information even if the information seems nonthreatening. Cambodian clients refuse to talk in order to feel safe. This is a form of defense mechanism that they used to survive under an authoritarian regime and to protect themselves against people they did not know.

Some of the more recent arrivals fear that they are unable to communicate with their host communities. Many also worry about family back home and about the difficulty of maintaining cultural and religious traditions. Recent refugees frequently are unemployed and lack the skills to gain employment. This is compounded by psychological and psychiatric problems related to war and violence. Clinicians reported examples of this in Bhutanese and Nepalese clients. 

Cambodian clients at the agency talked about violence in their pre-migration experiences as if the atrocities had just happened recently. In addition, the fears of being identified as an upper class Cambodian or working professional is still so intense that most of the people continue to use the language that was forced on them during the communist regime of the Khmer Rouge. For example, the multiplicity of the words for “to eat” in the Cambodian language, which are based on age and class, was removed by the communist regime and replaced with a single word that stripped off class and age subtexts. To this day some Cambodians still use this specific word for eating instead of the other culturally appropriate terms known in the language system pre-dating the Khmer Rouge regime. 

While many Cambodians cannot express why bad things happened to them during the war, they can describe some of the violent experiences in vivid detail. Some Cambodian clients at the agency, for example, still relive traumatic episodes from the past when triggered by a current event, which causes them paralysis and fear of being harmed again by some stranger or an authoritative figure (e.g., police, soldier, or guards). Some clients also expressed symptoms in physiological terms such as chronic headaches and body pain, sleeplessness, bad dreams, waking up with sweat and extreme anxiety, and loss of appetite.

##### Trauma, Stigma and Daily Challenges

Trauma plays a significant role in the lives of clients at the agency, but these difficulties are manifested in a manner that cannot be easily identified or understood. The agency’s clients often suffer in silence for fear or shame or because of a traumatic incident in their lives that caused them not to be able to open up about their psychological or psychiatric conditions.

In addition, many clients face cultural and linguistic challenges. They experience difficulty navigating through a system that is foreign to them, whether using public transportation or applying for public assistance. Clients face daily challenges such as getting around town, struggling with English, trying to find employment, getting housing and being a part of their community with people who share their experiences. They also were concerned with the lack of support systems, mistreatment from authorities, inability to fit into mainstream society, and violence in their neighborhoods. Those who have found work tend to work long hours with minimal pay and without benefits. If they accept cash, they receive no vacation, health insurance or retirement compensation. These challenges sometimes exacerbate what other symptoms they may have and can be a mental tipping point.

One other major issue with this population is their inability to express themselves in a manner that they view as dignified or without stigmatization. They do not know how to express their emotions and therefore tend to isolate themselves from people who could help them. The agency’s clients are a diverse group of people who share similar migration experiences, including their limited knowledge of Western diagnosis of psychological or psychiatric conditions. Some clients might have endured less psychological and emotional hardship before their resettlement in the U.S. Others might have been abused and mistreated and continue to cope with the challenges of living through trauma here in the U.S. Their children may also have potential difficulties because they may inherit their parents’ experiences of trauma. 

### 3.4. Challenges of Western Modalities vs. Eastern Philosophies

Inherent in this theme is the fundamental challenges of “Western Modalities versus Eastern Philosophies.” The Western-based modality of services, including the interpretation of mental illness and diagnosing using psychiatric labeling, leads to labeling clients unnecessarily; clients do not understand the relevance of such labels and treatments. For example, some diagnoses are made in order to be able to bill Medi-Cal for treatment. This type of intervention has term limits that do not necessarily coincide with how clients feel in regards to symptoms and management of symptoms (medical support). Often times, they feel lost as far as treatment is concerned, especially in regard to symptom management and resources.

Many of the agency’s clients continue to face challenges after their resettlement in the U.S. Some of them are major issues that could drive them into extreme desperation. One of the clinicians who spoke both Mandarin and Cantonese and carried a caseload of mostly Chinese-speaking clients remarked in his written notes that the lack of family support is a major issue for the majority of his clients. For example, he noted that one of his clients expressed a sense of desperation over not having his brother, sisters, or parents nearby after he learned that his oldest daughter was diagnosed with Down syndrome. This client was in his mid-fifties and struggling with depression himself. The clinician noted this client was struggling to understand the Western modality of a developmental disability and what it meant in the context of trying to care for his daughter. The clinician also noted that the client expressed psychological distress after receiving the news about his daughter’s diagnosis. The client regressed and experienced symptoms that included low energy, lack of motivation, and sleeplessness. In addition to his own struggle with psychological problems, the clinician noted that the client does not speak English. He noted that the client was facing many other challenges, such as utilizing public transportation, accessing public social services, and understanding the Western modality of developmental disabilities. These issues were also compounded by cultural values and beliefs of shame/stigma relating to psychological problems, mental illnesses, and disability issues in general. The clinician noted that the client felt lost and in desperation. He had no support systems because all his family members lived in China. 

Other clinicians noted that a good portion of their clients faced daily challenges. Some could not read or write in any language. Others could not see that there was a future being in this country. At the same time, most clients were still dealing with past experiences from their native countries, especially those individuals who were tortured or beaten. They have brought these experiences with them and are unable to express them in a manner that can help them. When they regress, some of the symptoms expressed by these clients make it apparent that they have suffered the experiences of war. They talk about their experiences as if the event recently happened. Some did not go through the event but talked as if they had. They said they felt generally not good because of their “sick minds.”

Southeast Asian refugees have endured many challenges that now they are dealing with the emotional consequences. Common themes that emerged from this group of Southeast Asians included the experience of violence in refugee camps and/or a prolonged stay in refugee camps, war/atrocities, starvation, persecution, diseases, and brutal regimes. Most did not feel the need to be open for fear that others might know of their psychological conditions. 

The agency’s clients are a diverse group of people comprising individuals from countries across the continent of Asia. They are from Afghanistan, China, Cambodia, Japan, Korea and Vietnam, as well as ethnic minority groups from Laos and Burma (now known as Myanmar) (e.g., Hmong, Karin and Mien). More recent groups come from Nepal and Bhutan. All these clients have in common is their shared experiences of war and trauma back home. Some were stripped of their former cultural identities, as in the case of the Chinese Cultural Revolution. In the case of atrocities in Cambodia, a significant portion of the population died under the Khmer Rouge regime as a result of execution, torture, disease, and starvation. The prolonged brutality of military regime in Burma also caused a major displacement of a large population. Burma’s military dictatorship that sought to ethnically cleanse certain minority groups from that country caused major displacement and death among various ethnic minority groups; most Burmese refugees lived in multiple refugee camps and were abused either by other ethnic groups or, like many Cambodians, were abused by those who were in charge of the camps. All these clients share a common history of trauma either in their native countries, during the migration process and/or the post-migration after resettlement in the U.S. 

### 3.5. Contrast with Other Immigrant/Refugee Groups

In many ways, the mental health and trauma described above are representative of immigrant and refugee group worldwide. For example, among the 1200 men, women, and children who sought refuge in Australia and forcibly relocated to the remote detention center on the pacific island of Nauru, many have suffered severe abuse, inhumane treatment and neglect [45]. Here they endured prison-like conditions, unnecessary delays for often life threatening medical conditions, sexual abuse by guards/overseers, overwhelming despair, and dire mental health problems. These refugees and asylum seekers, predominantly from Afghanistan, Bangladesh, Iran, Iraq, Kuwait, Pakistan, and Somalia, frequently have developed anxiety, inability to sleep, mood swing, prolonged depression, and short-term memory loss during their detention at Nauru facility. Children often wet their beds, suffer from nightmares, and engaged in disruptive and other troubling behaviors. Reports of intimidation, harassment, and violence are ubiquitous, with frequent suicidal attempts and self-harm. Similarly, there are clear parallels with the experiences of refugees fleeing conflict and persecution in the Middle East and seeking refuge in Europe. Shortfalls in humanitarian systems, limited livelihood opportunities, barriers to legal residency, illiteracy/language issues, inadequate educational opportunities, and rising xenophobic sentiment has resulted in dreadful mental health and physical conditions among these refugees as well. While many of the characteristic of immigrants and refugees are common regardless of their country of origin, some differences exist [46]. As mentioned in our analysis, the psychologic response of resettled Asian Americans and their families, differs in subtle ways from their counterparts in other cultures and regions of the world. Foremost, they are more hesitant to acknowledge and seek mental health services. 

## 4. Discussion

In order to understand Asian American community’s response to mental health problems, it is important to delve deep into the interconnectedness of health seeking-behaviors and cultural values as well as their experiences through migration and living in America [3,47]. Stigma/shame is intertwined with the ability to seek certain kinds of help. Stigma is a major barrier for Asian Americans and this is compounded with the belief that focuses on the drive to succeed and failure is not an option. The fact that such a low number of Asian Americans actually seek mental health services compared with the general U.S. population is, in part, attributable to stigma. Shame/stigma runs deep and is ingrained in Asian American cultural values, beliefs, and practices. The notion that children fail in school, for example, can shame the entire family and extend to parents as being inadequate role providers and children being disobedient. Asian Americans’ experience in America is not unique. They also faced discrimination dating back to the first waves of people in the late 1800s and early 1900s to arrive in America. For example, laws were passed to exclude land ownership or barring them from entering the U.S. The recent groups from Southeast Asia and elsewhere from the Asian continent are dealing with the psychological and emotional effects of the recent wars and are struggling to assimilate in the American mainstream community [48,49].

There are four periods in the immigrant/refugee experiences that can be identified, which include pre-migration, migration, encampment, and post-migration [22]. The above-mentioned questions have been addressed in this context. Each of these periods is said to have different stressors. Asian Americans’ lived experiences were explored in this process in order to gain a deeper understanding of their health-seeking behaviors, coping mechanisms, and trauma-related symptoms, including post-trauma stress disorder (PTSD).

Traumatic experience is not homogenous even within a specific region. Asian Americans consist of many diverse groups of people and their countries of origin spread across a continent from the Middle East to Insular Southeast Asia. Because of this diversity, their unique experiences can never be fully describe; however, there are some unique patterns that belong to specific regions, including mainland Southeast Asia, East Asia, and South Asia. The early arrivals’ experiences to America of people from Japan, Philippines, and China includes discrimination and segregation. Japanese were segregated and forced to live in temporary camps, for example. Encampment stressors are characterized by prolonged detainment in unsafe, over-crowded, and poorly sanitized temporary camps [22].

In addition, the pre-migration stressors of Southeast Asians were often extreme [50]. The Southeast Asians were subjected to government-sponsored intimidation and threats to their livelihood once the Communists gained power in their homelands. This includes death of family and friends, brutalization, loss of property and personal belongings associated with extensive and sustained warfare [22]. Once they were pressured to leave their native countries, some of the challenges that they faced include being separated from their loved ones and at the same time fleeing one’s home country under life-threatening conditions [22]. They faced ongoing trauma at various stages of migration. They never felt safe at any of the transitions in their journey. It was also common that Asian immigrants and refugees were assaulted by border guards while entering neighboring country to seek asylum abroad [51,52].

Among Southeast Asians, the Cambodians, Mien, and Hmong endured severe pre-migration trauma [53]. The Khmer Rouge led a bloody campaign of genocide to establish a Marxist agrarian society and free the country of any Western influence [54]. In their three-year reign, one-third of the population was killed owing to disease, mass execution, and poverty. People were forced to separate from their family members to work in labor camps. The Khmer Rouge targeted professionals and working classes with severe punishment, including beatings, starvation, and torture. The Mien and Hmong were recruited by the Central Investigation Agency (CIA) to help fight the war in Vietnam [55]. Their cultures are rooted in tribal agrarian and preliterate societies located in the mountain regions of Laos and other Southeast Asian countries. After the U.S. pulled out, they were tortured and ostracized by their own governments for assisting the Americans. The Burmese endured many years of brutality by the military regime. Among this diverse population group, the Korean group seems to have a unique migration experience. They have only two options if they wanted to come to America through sponsorship by a family member or applying to attend schools in the U.S. Once they are done with their study, they can choose to stay. This period of ambiguity is when they are most vulnerable to abuse. If they chose to stay after they complete their degrees, they have to apply for a green card, for example. Some choose to take this route and put themselves in a limbo while the U.S. government decides their fate. It is not uncommon for people to wait between five and ten years to hear about their residential status. In the meanwhile, their student visas expire and they would go underground instead of returning home. It is in this period of waiting that they are vulnerable to risks of being exploited by other groups. All of these groups of Asian Americans have some degree of trauma in their lives, whether it was in their pre-migration, migration or post-migration period. Their lived experiences demonstrate how trauma continues to affect their livelihood and well-being.

Trauma is a transgenerational process that overwhelms the community and the ability of family members to cope with life stressors. Examples include Asian Americans who arrived in the 19th century to work on the railroad projects and sugar plantations, Japanese encampment during World War II and Southeast Asian refugees who lived through the American/Vietnam war. A common bond of these groups were poor working conditions, discrimination, and poverty in their new homeland. These experiences had a profound impact on the children of the people who lived through these periods and many of them have not recovered from their traumatic experiences. The effects of conflict and violence on the mental health of a population have important implications for their overall well-being as well as livelihood. 

The undertaken observations suggest a psychological link of lived experiences with an event that happened five or more decades earlier. It is this collective experience that has caused many of them to vividly remember the past. Cultural trauma refers to a dramatic loss of identity and meaning, a fear in the social fabric that affect a group of people that have already achieved some degree of cohesion [11]. The Cultural Revolution is an example of a mass campaign to purge a culture of identity and values. Some Chinese Americans are still mourning that loss of identity and cultural values. Similarly, Cambodians juxtapose their past pre-migration experiences with the present. To this day, fear is manifested in many Cambodians, who still retain language of the Khmer Rouge to avoid being identified with the pre Khmer Rouge period.

Our exposé offers invaluable insight into the lives of those who experienced trauma and abuse owing to political conflict and cultural resettlement. These clients often suffer in isolation and are struggling to understand the Western modality of mental illness. Treatment, medication, and the like may offer them short-term relief; however, in order to be effective, long-term plans need to include support systems that are culturally sensitive and linguistically appropriate. The information on the agency’s clients offer a partial picture into difficult lives, the experiences clients have had, and the ongoing trauma they face in their neighborhoods. Some symptoms they expressed might have evolutionary roots that offer us some explanations as to why and how we as human beings react and respond to fear and trauma the way we do. We have evolved in ways that can be beneficial and also in ways that can be harmful.

Certain limitations should be noted when interpreting our observations. Foremost this was an ethnographic observational report by a single anthropologist and thus was limited in scope and time. Because it was not a formal research study, information was not collected on socio-demographics and migration related variables. Consequently, it was not possible to comment on selectiveness of the participants. Future observations including both qualitative and quantitative variables would offer a more complete picture of this population. Each region/geography has its own specific issues and challenges. Similarly, socio-economic status and educational levels may also affect how an individual views psychological or psychiatric problems and challenges in his/her life. Finally, the ability to be able to maneuver in this larger system of care and services can make a difference in how one functions in a community.

## 5. Conclusions

There are serious implications for social-workers and mental health professionals when working with or making observations pertaining to immigrant and refugees of API identity. Their expressions of experiences may differ from other vulnerable groups who have encountered high degrees of trauma, crime, violence, war, and poverty. In particular, there is a tendency to be less confrontational and more avoidant in behavior when it comes to addressing mental illness. Instead of looking for help, this population group tends to avoid using mental health services. The second implication is that this is a culturally diverse group of people without a common language with which they all can communicate; there is no single language or culture. In addition to values related to stigma and shame, immigrants/refugees as a group also tend to justify seeking mental health services by means of providing alternative explanations that involved physiological conditions rather than psychological or psychiatric symptoms. Finally, this population group appears to be less resistant to accessing mental health services from people who share similar cultural and ethnic backgrounds.

## References

[1] Asian-Pacific Institute on Gender-Based Violence (2015). Census Data & API Identities.

[2] Yoshihama M., Dabby C. (2014). Facts & Stats Report: Domestic Violence in Asian & Pacific Islander Homes.

[3] U.S. Department of Health & Human Services (2001). Reports of the Surgeon General, U.S. Public Health Service.

[4] He S. (2015). Asian American Mental Health Disparities and Cultural Psychiatry.

[5] Cheng A.W., Chang J., O’Brien J., Budgazad M.S., Tsai J. (2017). Model Minority Stereotype: Influence on Perceived Mental Health Needs of Asian Americans. J. Immigr. Minor. Health.

[6] Kwok J. (2013). Factors that influence the diagnoses of Asian Americans in mental health: An exploration. Perspect. Psychiatr. Care.

[7] Kim P.Y., Lee D. (2014). Internalized model minority myth, Asian values, and help-seeking attitudes among Asian American students. Cult. Divers. Ethn. Minor. Psychol..

[8] Atkinson J. (2002). Trauma Trails, Recreating Song Lines: The Transgenerational Effects of Trauma in Indigenous Australia.

[9] Chung S., Mikrogianakis A., Wales P.W., Dirks P., Shroff M., Singhal A., Grant V., Hancock B.J., Creery D., Atkinson J. (2011). Trauma association of Canada Pediatric Subcommittee National Pediatric Cervical Spine Evaluation Pathway: Consensus guidelines. J. Trauma.

[10] Atkinson J., Nelson J., Atkinson C., Purdie N., Dudgeon P., Walker R. (2010). Trauma, transgenerational transfer and effects on community wellbeing. Working Together: Aboriginal and Torres Strait Islander Mental Health and Wellbeing Principles and Practice.

[11] Schwarz E.D., Perry B.D. (1994). The post-traumatic response in children and adolescents. Psychiatr. Clin. N. Am..

[12] Courtois C.A., Ford J.D. (2013). Defining and understanding complex trauma and complex traumatic stress disorders. Treating Complex Traumatic Stress Disorders (Adults). Scientific Foundations and Therapeutic Models.

[13] Ho J. (2008). Community violence exposure of Southeast Asian American adolescents. J. Interpers. Violence.

[14] Ima K., Hohm C.F. (1991). Child maltreatment among Asian and Pacific Islander refugees and immigrants: The San Diego case. J. Interpers. Violence.

[15] Han M. (2006). Relationship Among Perceived Parental Trauma, Parental Attachment, and Sense of Coherence in Southeast Asian American College Students. J. Fam. Soc. Work.

[16] Ford J.D., Courtois C.A. (2014). Complex PTSD, affect dysregulation, and borderline personality disorder. Borderline Pers. Disord. Emot. Dysregul..

[17] Cloitre M., Garvert D.W., Brewin C.R., Bryant R.A., Maercker A. (2013). Evidence for proposed ICD-11 PTSD and complex PTSD: A latent profile analysis. Eur. J. Psychotraumatol..

[18] Barbara W.K.Y. (1997). The Social and Cultural Context of Adaptive Aging among Southeast Asian Elders. The Cultural Context of Aging Greenwood.

[19] Young-Eisendrath P. (1998). What Suffering Teaches.

[20] Palinkas L.A., Pickwell S.M. (1995). Acculturation as a risk factor for chronic disease among Cambodian refugees in the United States. Soc. Sci. Med..

[21] Mouanoutoua V.L., Brown L.G. (1995). Hopkins Symptom Checklist-25, Hmong version: A screening instrument for psychological distress. J. Pers. Assess..

[22] Abueg F.R., Chun K.M. (1996). Traumatization Stress among Asians and Asian Americans.

[23] 4 Flight from Indochina. http://www.unhcr.org/3ebf9bad0.pdf.

[24] Sack W.H., Him C., Dickason D. (1999). Twelve-year follow-up study of Khmer youths who suffered massive war trauma as children. J. Am. Acad. Child Adolesc. Psychiatry.

[25] Sack W.H., Clarke G.N., Seeley J. (1995). Posttraumatic stress disorder across two generations of Cambodian refugees. J. Am. Acad. Child Adolesc. Psychiatry.

[26] Savin D., Sack W.H., Clarke G.N., Meas N., Richart I. (1996). The Khmer Adolescent Project: III. A study of trauma from Thailand’s Site II refugee camp. J. Am. Acad. Child Adolesc. Psychiatry.

[27] Southeast Asia Resource Action Center (2015). Southeast Asia Resource Action Center: Annual Report.

[28] Sepic M. (2016). Brought as Kids, Possibly Deported as Adults, Several Cambodians await Federal Action.

[29] Ohman A., Mineka S. (2001). Fears, phobias, and preparedness: Toward an evolved module of fear and fear learning. Psychol. Rev..

[30] Keane T.M., Marshall A.D., Taft C.T. (2006). Posttraumatic stress disorder: Etiology, epidemiology, and treatment outcome. Annu. Rev. Clin. Psychol..

[31] Marks I., Tobena A. (1990). Learning and unlearning fear: A clinical and evolutionary perspective. Neurosci. Biobehav. Rev..

[32] Nesse R.M. (1990). Evolutionary explanations of emotions. Hum. Nat..

[33] Cantor C. (2005). Evolution and Posttraumatic Stress: Disorders of Vigilance and Defence.

[34] Bracha H.S., Ralston T.C., Matsukawa J.M., Williams A.E., Bracha A.S. (2004). Does “fight or flight” need updating?. Psychosomatics.

[35] Galliano G., Noble L., Travis L., Puechl C. (1993). Victim Reactions during Rape/Sexual Assault. J. Interpers. Violence.

[36] Bados A., Toribio L., Garcia-Grau E. (2008). Traumatic events and tonic immobility. Span. J. Psychol..

[37] Lerner J.S., Keltner D. (2001). Fear, anger, and risk. J. Pers. Soc. Psychol..

[38] Sylvers P., Lilienfeld S.O., LaPrairie J.L. (2011). Differences between trait fear and trait anxiety: Implications for psychopathology. Clin. Psychol. Rev..

[39] Nicassio P.M. (1985). The Psychosocial Adjustment of the Southeast Asian Refugee An Overview of Empirical Findings and Theoretical Models. J. Cross-Cult. Psychol..

[40] Schwerdtfeger K.L., Goff B.S. (2007). Intergenerational transmission of trauma: Exploring mother-infant prenatal attachment. J. Trauma. Stress.

[41] Stewart D.W., Shamdasani P.N., Rook D.W. (2007). Focus Groups: Theory and Practice.

[42] Seymour J. (2012). Combined qualitative and quantitative research designs. Curr. Opin. Support. Palliat. Care.

[43] Post-Traumatic Stress Disorder among Adults. https://www.nimh.nih.gov/health/statistics/prevalence/post-traumatic-stress-disorder-among-adults.shtml.

[44] Song S.J., Subica A., Kaplan C., Tol W., de Jong J. (2017). Predicting the Mental Health and Functioning of Torture Survivors. J. Nerv. Ment. Dis..

[45] Amnesty International Canada (2016). Australia: Appalling Abuse, Neglect of Refugees on Nauru.

[46] Refugee Council of Australia (2016). Australia’s Response to a World in Crisis.

[47] Sudhinaraset M., Ling I., To T.M., Melo J., Quach T. (2017). Dreams deferred: Contextualizing the health and psychosocial needs of undocumented Asian and Pacific Islander young adults in Northern California. Soc. Sci. Med..

[48] Wong E.C., Marshall G.N., Schell T.L., Berthold S.M., Hambarsoomians K. (2015). Characterizing the Mental Health Care of U.S. Cambodian Refugees. Psychiatr. Serv..

[49] Thikeo M., Florin P., Ng C. (2015). Help Seeking Attitudes Among Cambodian and Laotian Refugees: Implications for Public Mental Health Approaches. J. Immigr. Minor. Health.

[50] Anderson J., Moeschberger M., Chen M.S., Kunn P., Wewers M.E., Guthrie R. (1993). An acculturation scale for Southeast Asians. Soc. Psychiatry Psychiatr. Epidemiol..

[51] Casella A. (1989). The Refugees from Vietnam: Rethinking the Issue.

[52] Barry W. (1981). The Refused: The Agony of the Indochina Refugees.

[53] Haing N. (1987). A Cambodian Odyssey.

[54] Hinton A. (2011). Genocide, categorical certainty, and the truth: Questions from the Khmer Rouge Tribunal. J. Anal. Psychol..

[55] Leary W.M. (2008). CIA Air Operations in Laos, 1955–1974. Supporting the “Secret War”.

